# Technology Evolution of China’s Export of Renewable Energy Products

**DOI:** 10.3390/ijerph15081782

**Published:** 2018-08-19

**Authors:** Xuping Cao, Aroskar Rajarshi, Juxi Tong

**Affiliations:** 1 School of Economics and Management, Changshu Institute of Technology, Changshu 215500, China; juxit@cslg.edu.cn; 2 School of Business, University of Wisconsin-Eau Claire, Eau Claire, WI 54702, USA; AROSKAR@uwec.edu; 3 Suzhou Agricultural Modernization Research Center, Changshu Institute of Technology, Changshu 215500, China

**Keywords:** technical complexity index, renewable energy products, China

## Abstract

With the increasing scarcity of traditional energy sources, global warming and environmental degradation, the increased use of renewable energy (RE) has become an effective path for sustainable development. Therefore, countries are paying more and more attention to the development of the RE industry, and the world trade in renewable energy products (REPs) is developing rapidly. First of all, this paper defines REPs, refines the scope of REPs, and proposes the “Equalization Technology Classification” method for the technology classification of REPs. Second, based on the United Nations Comtrade (COMTRADE) data, the export technology structure of China’s REPs from 2007 to 2016 was empirically measured. Finally, a comparative study was conducted on the renewable energy product (REP) export technologies of major REP exporting countries (or regions) in the world. We found that (1) China’s exports of REPs are mainly medium-high and medium technical complexity products, and that there are few high technical complexity products; (2) the export technology structure of China’s REPs is deteriorating, and its overall technical level is in the middle of the global industrial value chain. The export technology of China’s REPs has a gap compared with that of Denmark, Hong Kong China, and Singapore; (3) the technological competition of the world’s REPs is becoming increasingly fierce. The growth rates of REP technologies in South Korea, Japan, and Malaysia’s REPs are significantly higher than that of China.

## 1. Introduction

Renewable energy (RE) is a non-traditional energy source that includes solar energy, wind energy, biomass energy, and nuclear fusion energy [1]. It is widely available, renewable, low-carbon and has minimal environmental impact [2]. With the increasing scarcity of traditional energy sources, global warming, and environmental degradation, the increased use of RE has become an effective path for sustainable development [3,4]. At present, RE accounts for a small proportion of the world’s energy use [5]. However, with the continuous advancement of the RE utilization technology, it is an irresistible trend to replace traditional energy sources with RE [6,7]. With a large population, China is one of the world’s largest energy consumers [8]. As early as 2010, the RE industry was listed by China as one of the seven strategic emerging industries in the country [9]. In 2016, China’s “13th Five-Year Plan for RE Development” proposed to accelerate the establishment of a clean, low-carbon, safe and efficient modern energy system, and to achieve the goal of non-fossil energy accounting for 15% of primary energy consumption by 2020 [10].

In recent years, China’s RE industry has developed rapidly. Statistics show that China has become the world’s largest RE investor, owner of RE vehicles, and REP producer and consumer country [11]. Correspondingly, the export of REPs has also developed rapidly. According to COMTRADE data, China’s REP exports in 2016 reached $83.40 billion, accounting for 24.31% of the world’s export share, ranking first in the world [12]. 

However, China’s economy has entered a new stage of “coming to a quality revolution made in China”, and the quality of export commodities is more important than quantity [13]. In recent years, China has actively integrated into the process of economic globalization by taking advantage of its low labor resources, and many industries have already led the world in export volume [14]. Economic theory and international development experience show that sustainable economic growth is inseparable from the continuous optimization of export technology structure [15]. Fan et al. (2009) [16] found that the proportion of low value-added products in China’s export structure showed a downward trend, and medium-level products gradually became the main export products. It is concerningas to whether the evolutionary trend of China’s REP exports is the same as that of China’s export technology. To this end, this paper will empirically measure the changes in export technology of China’s REPs.

Previous literature studies have focused on the export competitiveness, export challenges, and export technology measures of REPs [17]. Wei et al. (2016) [18] found that RE equipment is China’s second largest export-oriented environmental product, and that its export share is second only to wastewater treatment products. Solar photovoltaic cellsare one of the world’s important export REPs. Zhao et al. (2017) [19] found that most of the world’s solar photovoltaic cell exporters are located in East Asia and Southeast Asia, and that the international trade intensity of solar photovoltaic cell is growing. Fu et al. (2013) [20] found that the export of China’s REPs to the US, EU, and Japan markets showed a rapid growth. However, Fu et al. (2013) [20] only analyzed the export of 4-digit Harmonized Commodity Description and Coding System (HS) coded products. Four-digit HS-coded products contain many non-renewable energy product categories, and the definition of REPs is not precise enough. This paper defines the scope of REPs from the 6-digit HS code, and partially eliminates or supplements the Fu et al. (2013) [20] classified products. Additionally, there is no clear authoritative definition of REPs. This study defines REPs as equipment and related products that provide services for the efficient use of RE, and the development of RE industries.

Although China’s REPs have achieved good export performance, the country still faces many problems or challenges, such as lack of high technology, unsustainable government subsidy policies, and unpredictable global trade environment [21,22,23,24,25]. Most of the global RE dispute cases since 2007 are related to China [26]. For example, the solar photovoltaic dispute between China and USA, since 2012 has had a serious negative impact on China’s solar photovoltaic industry and its exports [27,28]. This fully exposes the overcapacity of low technology products in China’s RE industry [29]. This article will focus on the export technology structure of China’s REPs.

For the measurement of export technology, Guan et al. (2002) [30] proposed the technology added value method. Lall et al. (2006) [31] proposed a complex index method, and Du et al. (2007) [32] revised the method. Hausmann et al. (2005) [33] proposed the use of “product-relevant income levels” (*PRODY*) to determine the level of product labor productivity, also known as technical complexity index. What these methods have in common is that they first determine the technical level of a single product, then they calculate the overall technical level of the economy, and assign the technology content of the product to the weighted sum of the income levels of countries (or regions). The difference between these methods is the assignment weight. The valuation weights of Guan et al. (2002) [30] and Lall et al. (2006) [31] are the world share of various products exported by various countries. Du et al. (2007) [32] revised the weight of the method to the world share of various types of products produced by various countries. The valuation weights of Hausmann et al. (2005) [33] are the export comparative advantage index after the standardization of various products in various countries. In comparison, the application of the technique complexity method of Hausmann et al. (2005) [33] is more common, and the research data is more acquirable. These application areas involve cultural and creative industries, manufacturing, agriculture, etc., but there is a lack of research on REPs export technology [34,35,36]. To this end, this paper chooses the technical complexity index of Hausmann et al. (2005) [33] to empirically measure the dynamic changes of China’s REP export technology. The Hausmann et al. (2005) [33] method does not classify *PRODY* values. This study proposes a method called “Equalization Technology Classification” that divides all REPs into five technical levels: high, medium-high, medium, medium-low, and low according to the *PRODY* value. This method facilitates a clearer REP technology analysis and international comparison.

Different classification criteria for *PRODY* values will have different effects on the conclusions of the study. The main classification methods of the previous literature are the “Experience Sorting Method” of Tang (2012) [37], the “Technical Fixed Classification” of Zhu et al. (2009) [38], and the “Optimal Segmentation Method” of Wei (2015) [15]. The method of Tang (2012) [37] classifies the *PRODY* values according to the author’s experience, ensuring that the technology classifications are as normal as possible, and the classification results of different scholars may be different. The method of Zhu et al. (2009) [38] ignores the fact that technology changes over time. The method of Wei (2015) [15] sorts *PRODY* data, and then determines the number of categories according to the needs, which are also likely to cause people to subjectively change the technology differences between samples. To this end, the “Equilibrium Technology Method” proposed in this paper emphasizes objectivity and will avoid the classification results being influenced by time change and human experience.

The review shows that technological innovation is one of the important factors affecting the sustainable development of China’s RE industry. However, there is little research literature on the structural changes in export technologies for REPs. Moreover, the definition and technology classification of REPs need to be further improved [20]. To this end, this paper is based on previous literature research [20], to first refine the scope of REPs further. Then, this paper uses the technical complexity index to empirically measure the dynamic changes of China’s REP export technology. In order to ensure the objectivity of product technical complexity classification, this paper proposes the “Equalization Technology Classification” method.

## 2. Methods and Data

### 2.1. Methods

The export complexity method proposed by Hausmann et al. (2005) [33] uses international trade data to replace hard-to-find research and development (R&D) data for various types of products in countries (or regions) around the world. The basic assumption of this method is that the more technical a class of products that are being exported from high-income countries, the higher the technical complexity of the product. This method does not consider other factors such as trade friction and intervention [39]. In the global manufacturing value chain, developed countries are generally in the process of high value-added value such as R&D design, brand, and key parts production, while developing countries are more involved in low value-added links such as raw material supply and assembly. The status of countries in the global value chain will be reflected in the technological structure of the products that they export. This method combines the per capita income of countries (or regions) with exports. The technological content of the export products of these countries (or regions) in the global value chain can be measured. The method used in this paper consisted of three steps: calculating the technical complexity of various REPs, classifying the technical complexity of different REPs, and calculating the overall technical level of each country (or region).

(1) The equation for the technical complexity of REP export. The *PRODY_k_* is the export technical complexity of the category *k* export REPs at the world level. The equation [33] for *PRODY_k_* is:

(1) $$PRODY_{k}=\sum_{j}\frac{x_{jk}/X_{j}}{\sum_{j}x_{jk}/X_{j}}\times Y_{j}\;$$

The notations in Equation (1) and their meanings are as follows:

*j*  the *j*th REPs exporting country (or region)
*k*  the category of export REPs
*X_j_*  the total exports of all REPs in the country (or region) *j*
*x_jk_*  the export value of category *k* REPs of country (or region) *j*
  of RADE d the COMTRADE database
*Y_j_*  the per capita gross domestic product (GDP) of country (or region) *j*
*PRODY_k_*  the export technical complexity of the category *k* export REPs at the world level

(2) The principle of the “Equalization Technology Classification” method: the basic principle of this method is to ensure that the *PRODY* value difference of adjacent technology grade products is equal, and that there is no limit to the number of products that are owned by each grade.

First, the calculated *PRODY* values of *n* type REPs were arranged from smallest to largest, into an ordered sample (*t*_1_, *t*_2_, *t*_3_, *...*, *t_n_*), where *t*_1_ is the smallest and *t_n_* is the largest.

Second, it was assumed that the REP technology was divided into *m* grades, and that the *PRODY* value difference of the adjacent technology grade products was *D*. The equation for *D* is:

(2) $$D=\frac{t_{n}-t_{1}}{m}\;$$

Finally, the technology classification criteria for REPs were calculated. The standards for the 1, 2, 3, ..., *m* levels of REPs were *PRODY* ≤ *t*_1_ + *D*, *t*_1_ + *D* < *PRODY* ≤ *t*_1_ + 2*D*, *t*_1_ + 2*D* < *PRODY* ≤ *t*_1_ + 3*D*, ... *t_n_* – *D* < *PRODY*. This method can determine the technology classification standards of all REPs in the world in a certain period of time. According to this standard, it is possible to clearly know how many high technical REPs are exported by a country (or region).

(3) The calculation method of overall export technical level (which we call *EXPY*) of REPs. Assume that the overall export technical level of REPs in country (or region) *j* is *EXPY_j_*, and its equation [35] is:

(3) $$EXPY_{j}=\sum_{k}\frac{x_{jk}}{X_{j}}\times PRODY_{k}\;$$

The calculated *EXPY* values are mainly used to compare the overall technical level of REPs in countries (or regions).

### 2.2. Data

#### 2.2.1. Scope Definition for Reps

At present, there is no uniform authority defining standard for the range of REPs. Fu et al. (2013) [20] divides REPs into five categories: nuclear energy, wind energy, solar energy, biomass energy, and smart grid, and 14 subdivided 4-digit HS code products. However, some 4-digit code connotation 6-digit products do not belong to the energy industry, while smart grids are mainly traditional power facilities. Based on an in-depth study of the HS code and the China Import and Export Tariff, this study revised the classification of Fu et al. (2013). This paper divided REPs into fourcategories: nuclear energy, wind energy, solar energy, and biomass energy. Each category contained many sub-categories. The detailed categories of REPs and their HS codes are shown in Table 1.

As can be seen from Table 1, the five categories of products contained a total of 28 categories of sub-product categories. Since some of the 4-digit code products contained only a part of the 6-digit code products belonging to REPs, the sub-product categories in Table 1 included both HS 4-digit and HS 6-digit code products. 

#### 2.2.2. Sample Selection and Data Source

This article selected Austria, Belgium, Brazil, Canada, China, Hong Kong China, Czech Republic, Denmark, Finland, France, Germany, Hungary, India, Italy, Japan, Malaysia, Mexico, Netherlands, Poland, South Korea, Romania, Russia, Singapore, Spain, Sweden, Switzerland, Thailand, the United Kingdom, and USA as analytical samples. These 29 samples are the world’s major REPs exporting countries (or regions) from 2007 to 2016 (Table A1). The COMTRADE data showed that their REPs exports accounted for more than 90% of the world’s total, and they were highly representative samples [12]. The per capita gross domestic product (GDP) of each country (region) was derived from the World Bank database and was converted to purchasing power parity (PPP) (Table A2) [40]. The PPP in this article used the “Constant 2011 International $” standard. In order to ensure the consistency in research data, the export data of this paper were all from the COMTRADE database, and the commodity code adopted the HS2007 standard.

## 3. Results

### 3.1. Division of Technology Structure Standards of Various Products

Using Equation (1), the annual average of *PRODY* (Table A3) for REPs from 2007 to 2016 was calculated. Then, the *PRODY* values were divided into five grades by using the “Equalization Technique Classification” method. The result is a classification of REPs into high technical complexity products ($36,858 < *PRODY*), medium-high technical complexity products ($30,550 < *PRODY* ≤ $36,858), medium technical complexity products ($32,653 < *PRODY* ≤ $34,755), medium-low technical complexity products ($32,873 < *PRODY* ≤ $32,653), and low technical complexity products ($28,448 < *PRODY* ≤ $30,550) (Table 2). The technology structure distribution of the world’sREPs is shown in Table 2.

As can be seen from Table 2, the number of high technical complexity products was the highest, up to eight. Products with medium-high, medium-low technical complexity each had seven categories. There were four kinds of technical complexity products, and only two kinds of low technical complexity products.

### 3.2. Dynamic Distribution of Technology Structure of China’s REPs

According to the classification in Table 2, the export shares of different technology classifications of China’s REPs from 2007 to 2016 are shown in Table 3.

As can be seen from Table 3, China’s REP exports are dominated by medium-high technical complexity products. From 2007 to 2016, the average annual export share of medium-high technical complexity products accounted for 50.28%. However, the average annual export share of China’s high technical complex REPs was only 4.58% (Table 3). Additionally, the export technology structure of China’s REPs is deteriorating. From the trend of change, the export shares of high and medium-high technical complex products showed a significant decline. The proportion of cumulative exports of the two types of products decreased from 57.88% in 2007 to 45.95%, a drop of 20.61% (Table 3). In contrast, the export share of medium and medium-low technical complexity products is gradually increasing. In 2016, China’s REP export technical complexity below the medium-high level accounted for 54.04% (Table 3). The higher the technical complexity index of export REPs, the greater the added value of their exports. Therefore, it is urgent to optimize the export technology structure of China’s REPs.

### 3.3. International Comparison of Export Technologies for REPs

Using Equation (3), the *EXPY*s for REPs in 29 countries (or regions) from 2007 to 2016 were calculated. This paper selects 14 countries (or regions) with the highest annual average *EXPY* value for comparative study. These countries (or regions) are Hong Kong China, Singapore, the Netherlands, Switzerland, Austria, China, Malaysia, Finland, USA, South Korea, Japan, Sweden, Germany, and Denmark (Table 4). The comparison data of the *EXPY* changes of REPs in 14 countries (or regions) from 2007 to 2016 are shown in Table 4.

As can be seen from Table 4, (1) In 2007–2016, the annual average of *EXPY* of REPs in Denmark ranked first in the world, and Hong Kong China ranked second. The difference in *EXPY* between the remaining 12 countries was not large. In Denmark, for example, the reason for its high *EXPY* value is that its high technical complexity index product 850231 (wind power generation equipment) has a high proportion of exports and is highly competitive in the world. In 2016, the export volume of Danish 850231 products reached $3.17 billion, accounting for 42.99% of the total export share of 29 countries (or regions) [5]. Denmark is a veritable wind power kingdom, with the highest per capita RE consumption index, nearly 5000 wind power plants, and the strongest wind power technology. In 2015, the annual average coverage of wind power in Denmark reached 42% [41]. Denmark plays a pivotal role in the international wind power and equipment export market.

(2) From 2007 to 2016, the overall technology of China’s REPs is at a medium technical complexity index level, and there is still a certain gap compared with Denmark, Hong Kong China, and Singapore. China’s main export product categories 854140 (solar cells) and 850440 (inverters) belong to the medium-high technical complexity index products, 940540 (solar power station), and 854370 (solar lighting equipment) belong to the category of medium technical complexity index products.

(3) According to the trend of change, the *EXPY* of China’s REPs has shown a rapid growth trend. The *EXPY* value increased from $33,610 in 2007 to $36,378 in 2016 (Table 4), indicating that the overall technical level of China’s REPs is constantly improving. It is worth noting that the *EXPY* values of all major REPs exporting countries (or regions) are growing, but the growth rates of *EXPY* values in South Korea, Japan, and Malaysia’s REPs are significantly higher than that of China. In 2016, the *EXPY* values of these three countries exceeded China’s. These phenomena fully demonstrate that the technological competition of the world’s REPs is increasingly fierce.

## 4. Discussions

(1) This paper puts forward some inspirational suggestions to promote the technological progress of China’s RE industry. First, the low proportion of high technical complex exports restricts the overall technical level of China’s REPs. China’s REP manufacturers need to abandon short-term market interests, strengthen investment in talent and technology research, and strive to enhance its position in the global RE industry value chain. Second, the form of export trade is too singular and easily causes international trade friction. Therefore, China’s REP producers should actively expand cooperation with leading technology countries, such as the docking of RE technology standards, cooperative research and development (R&D) of RE equipment, exchange of REP technology talents, etc. Third, China’s REP producers need more “going out”, making full use of foreign resources and technology. 

(2) The definition of the scope of REPs has a greater impact on the conclusions of the study. The REPs belong to the category of environmental products. At present, the authoritative definition of environmental products has only been discussed by two international organizations, the Asia-Pacific Economic Cooperation (APEC) and the Organization for Economic Co-operation and Development (OECD), but the differences between the two standards are still large. In the future, this research field deserves further exploration.

(3) Different classification methods of *PRODY* values will also affect the conclusion of the study. This study uses the “Equalization Technology Classification” method, and if other methods are used, the research conclusions will be different. Therefore, the classification of technology structure is worthy of further exploration.

(4) The technical complexity index also has limitations. For example, the processing trade factor and the implementation of the technology export restriction policy are not considered. Therefore, the research conclusion is only used as a reference for decisionmaking. Therefore, future improvements and application studies on this method are worth exploring.

## 5. Conclusions

The possible innovation of this paper is to define REPs, to revise the scope of REPs, and to propose the “Equalization Technology Classification” method for technical complexity index classification. This paper also expands upon the application of the technical complexity index in the field of REP. The technical complexity index has been used to empirically measure China’s REP export technology structure from 2007 to 2016. 

The study found that China’s REP exports are dominated by medium-high and medium technical complexity products. The proportion of high technical complex export REPs is very low, and the overall export technology structure is deteriorating. In 2016, China’s REP export technical complexity below the medium-high level accounted for 54.04%. The overall technical level of China’s export REPs is at the middle of the global industrial value chain.

Additionally, Denmark has taken the lead in global REP technology by virtue of its wind energy products. The export technology of China’s REPs has a certain gap compared with that of Denmark, Hong Kong China and Singapore. The REP technologies of all major REP exporting countries (or regions) are growing, but the growth rates of REP technologies in South Korea, Japan, and Malaysia’s REPs are significantly higher than that of China. In short, the technological competition of the world’s REPs is increasingly fierce.

## Figures and Tables

**Table 1:** The categories of renewable energy products (REPs) and their Harmonized Commodity Description and Coding System (HS) codes.

Product Category	Commodity Code	Commodity Descriptions
Nuclear energy products	8401	Nuclear reactor; unirradiated fuel element (heat release element) of nuclear reactor; isotope separation machine and device; other internal components
	8402	Steam boiler (except for hot water boilers for central heating that can generate low-pressure steam); superheated water boiler
	8404	Boiler auxiliary equipment (for example, economizer, superheater, ash remover, gas recovery); condenser for steam or steam power plant
	841950	Nuclear reactor dedicated heat exchanger; steam generator (dedicated to the generation of heat in a nuclear reactor into steam)
Wind energy products	841239	Other pneumatic power units
	850231	Wind power equipment
	850300	Wind power equipment parts
	903289	Wind power equipment controller
	722840	Forging tool round steel (mainly used for wind energy)
	853710	Wind energy controller
	841480	Air compressor accessories (air duct)
	841490	Air compressor parts (impellers, blades, etc.)
	392099	Plastic sound board on the wind blade
Solar products	854140	Solar cell; light emitting diode; other photosensitive semiconductor device
	850239	Generator set that relies on renewable energy (RE) to produce electricity
	841919	Solar water heaters
	850440	Solar inverter, converter, regulated power supply
	854370	Solar power station
	850720	Lead-acid cell for solar energy
	940540	Solar-related lighting device
	901390	Solar heliostat parts
	280461	Polysilicon
Biomass energy products	440130	Sawdust, waste and other biomass
	841620	Gas burners, burners, etc.
	850220	Natural gas power generation unit, generator
	8405	Gas, acetylene and similar hydrolyzed gas generators
	847930	Granulator; wood extruder; biomass mill, etc.
	840790	Biogas engine

Note: The data comes fromthe United Nations Comtrade (COMTRADE) database [12], and the product description is streamlined.

**Table 2:** The technology structure distribution of the world’s REPs.

Technical Complexity Classification	Classified Standard/$	Product HS Code
High technicalcomplexity products	36,858 < *PRODY*	841620, 722840, 850239, 850231, 392099, 280461, 850220, 841239
Medium-high technicalcomplexity products	34,755 < *PRODY* ≤ 36,858	841950, 440130, 854140, 847930, 841490, 8405, 850440
Medium technicalcomplexity products	32,653 < *PRODY* ≤ 34,755	841480, 901390, 940540, 854370
Medium-low technical complexity products	30,550 < *PRODY* ≤ 32,653	8404, 850300, 850720, 8401, 841919, 903289, 853710
Low technicalcomplexity products	28,448 < *PRODY* ≤ 30,550	840790, 8402

Note: The raw data of the calculation results are from the COMTRADE database.

**Table 3:** Dynamic distribution of export technology structure of China’s REPs.

Years	High-Tech Complexity Ratio/%	Medium-High Technical Complexity Ratio/%	Medium-Tech Complexity Ratio/%	Medium-Low Technical Complexity Ratio/%	Low-Tech Complexity Ratio/%
2007	6.28	51.60	22.85	15.90	3.37
2008	6.11	51.54	21.04	15.91	5.39
2009	4.68	52.42	20.15	16.21	6.55
2010	3.61	60.27	18.84	13.54	3.73
2011	4.32	55.86	21.80	13.97	4.05
2012	4.88	49.69	24.75	16.85	3.84
2013	4.90	46.90	28.04	16.85	3.31
2014	4.16	46.24	30.03	16.63	2.94
2015	3.48	45.74	31.70	16.51	2.58
2016	3.37	42.58	33.71	17.71	2.63
Annual Average	4.58	50.28	25.29	16.01	3.84

Note: The raw data of the calculation results are from the COMTRADE database.

**Table 4:** Changes in overall export technical level (*EXPY*) values of REPs in 14 countries (or regions).

Years	*EXPY* Value of REPs/$													
	Denmark	Hong Kong China	Singapore	Netherlands	Switzerland	Austria	China	Malaysia	Finland	USA	South Korea	Japan	Sweden	Germany
2007	32,824	33,647	33,483	33,613	34,133	34,097	33,610	33,396	34,077	33,714	32,995	33,505	33,643	33,428
2008	32,572	34,160	34,226	34,204	34,756	34,496	33,557	32,954	34,081	34,119	33,259	33,342	34,310	33,485
2009	31,665	32,810	32,848	32,761	33,534	32,831	32,106	31,486	32,905	32,834	31,722	32,073	33,103	32,232
2010	35,260	34,849	33,690	33,532	34,006	33,742	33,471	32,296	34,198	33,870	33,365	33,196	33,378	33,295
2011	36,908	35,340	34,700	34,222	34,259	34,556	34,479	33,846	34,713	34,179	34,406	34,174	34,468	34,018
2012	35,994	35,583	34,871	34,571	34,319	34,701	34,815	34,625	34,263	34,306	34,456	34,593	34,611	34,491
2013	37,006	36,087	35,035	34,807	34,548	34,432	35,170	35,153	34,548	34,383	34,992	34,747	34,242	34,685
2014	37,413	36,616	35,646	35,513	34,937	34,751	35,554	35,851	34,797	34,993	35,793	35,287	34,720	35,096
2015	38,652	37,105	36,694	36,075	35,288	35,529	36,110	37,189	35,253	35,409	36,150	35,945	34,724	35,762
2016	39,335	37,804	37,533	36,604	35,708	36,159	36,378	38,053	35,592	36,024	36,633	36,491	35,636	36,219
Annual Average	35,763	35,400	34,873	34,590	34,549	34,529	34,525	34,485	34,443	34,383	34,377	34,335	34,283	34,271

Note: The raw data of the calculation results are from the COMTRADE database.

## Abbreviations

The following abbreviations are used in this manuscript:

RE	Renewable energy
REPs	Renewable energy products
REP	Renewable energy product
COMTRADE	United Nations Comtrade
R&D	Research and development
PPP	Purchasing power parity
GDP	Gross domestic product
*PRODY*	Product-relevant income levels
*EXPY*	Overall export technical level
HS	Harmonized Commodity Description and Coding System
OECD	The Organisation for Economic Co-operation and Development
APEC	Asia-Pacific Economic Cooperation

## Appendix A

**Table A1:** The world’s major renewable energy products (REPs) exporters from 2007 to 2016.

Country (or Region)	The Annual Average of Export Value/$100 Million
China	838.99
Germany	374.22
USA	321.62
Japan	205.69
South Korea	136.30
Mexico	124.42
Hong Kong China	108.89
Italy	93.78
Malaysia	78.24
Thailand	72.62
Netherlands	69.35
France	65.52
Singapore	65.50
United Kingdom	63.58
Hungary	61.24
Denmark	51.54
Spain	42.99
Canada	42.33
Poland	40.93
Belgium	39.47
Austria	37.34
Romania	36.14
Czech Republic	34.39
Switzerland	32.43
Sweden	30.07
India	28.58
Russia	25.76
Finland	19.35
Brazil	10.80

Note: The data come fromthe COMTRADE database.

**Table A2:** The gross domestic product (GDP) ^1^ per capita of major exporters, based on purchasing power parity (PPP) ^2^ from 2007 to 2016.

Country (or Region)	GDP Per Capita/$									
	2007	2008	2009	2010	2011	2012	2013	2014	2015	2016
Austria	43,878	44,418	42,619	43,336	44,453	44,552	44,303	44,345	44,354	44,491
Belgium	41,623	41,619	40,356	41,086	41,249	41,046	40,928	41,384	41,723	42,095
Brazil	13,271	13,806	13,653	14,539	14,973	15,118	15,430	15,371	14,666	14,024
Canada	41,647	41,611	39,924	40,699	41,565	41,795	42,339	43,079	43,149	43,238
China	7285	7948	8652	9526	10,384	11,146	11,951	12,759	13,570	14,399
Hong Kong China	45,937	46,635	45,390	48,108	50,086	50,378	51,732	52,789	53,595	54,354
Czech Republic	28,844	29,373	27,804	28,353	28,797	28,527	28,380	29,120	30,605	31,339
Denmark	46,374	45,866	43,383	43,998	44,403	44,337	44,564	45,057	45,459	45,991
Finland	42,467	42,575	38,868	39,848	40,684	39,913	39,428	39,018	38,942	39,659
France	37,772	37,635	36,341	36,872	37,457	37,345	37,367	37,531	37,766	38,061
Germany	40,474	40,989	38,784	40,429	42,693	42,822	42,914	43,561	43,938	44,357
Hungary	23,492	23,734	22,202	22,404	22,841	22,582	23,119	24,161	25,034	25,664
India	3699	3787	4050	4405	4636	4828	5074	5390	5754	6093
Italy	38,612	37,954	35,710	36,201	36,347	35,228	34,220	33,946	34,302	34,655
Japan	36,697	36,278	34,317	35,750	35,775	36,368	37,149	37,337	37,883	38,283
South Korea	28,014	28,588	28,643	30,352	31,229	31,777	32,549	33,426	34,178	34,986
Malaysia	20,685	20,989	20,092	21,107	21,819	22,591	23,224	24,195	25,002	25,669
Mexico	16,044	16,008	15,012	15,535	15,923	16,324	16,316	16,460	16,672	16,832
Netherlands	46,528	47,134	45,126	45,525	46,067	45,411	45,191	45,668	46,494	47,270
Poland	19,563	20,392	20,953	21,771	22,851	23,218	23,555	24,347	25,300	26,036
Romania	17,277	19,053	17,855	17,818	18,095	18,292	19,009	19,667	20,545	21,615
Russia	22,799	24,006	22,122	23,108	24,310	25,156	25,551	25,285	24,517	24,417
Singapore	68,423	66,037	63,688	72,105	75,013	76,029	78,549	80,305	80,892	81,443
Spain	34,330	34,164	32,653	32,507	32,068	31,109	30,679	31,195	32,291	33,320
Sweden	44,051	43,466	40,863	42,943	43,755	43,308	43,476	44,168	45,679	46,568
Switzerland	56,269	56,756	54,806	55,866	56,184	56,150	56,536	57,218	57,264	57,428
Thailand	12,607	12,757	12,605	13,487	13,535	14,448	14,778	14,853	15,237	15,683
United Kingdom	38,384	37,903	36,042	36,367	36,608	36,893	37,399	38,252	38,839	39,309
USA	51,011	50,384	48,558	49,373	49,791	50,520	51,008	51,932	53,029	53,445

Note: ^1^ The data come from the World Bank; ^2^ The PPP uses the “Constant 2011 International $” standard.

**Table A3:** The annual average of *PRODY* for REPs from 2007 to 2016.

HS Coding	The Annual Average of *PRODY*/$
8401	32,035
8402	28,716
8404	30,879
8405	36,511
280461	38,348
392099	37,693
440130	35,256
722840	36,971
840790	28,448
841239	38,961
841480	33,036
841490	36,284
841620	36,870
841919	32,095
841950	34,933
847930	36,014
850220	38,468
850231	37,370
850239	37,145
850300	31,244
850440	36,839
850720	31,968
853710	32,403
854140	35,478
854370	34,347
901390	33,048
903289	32,280
940540	33,660

Note: The raw data of the calculation results are from the COMTRADE database.
